# Chromosome analysis of foetal tissue from 1903 spontaneous abortion patients in 5 regions of China: a retrospective multicentre study

**DOI:** 10.1186/s12884-023-06108-0

**Published:** 2023-11-25

**Authors:** Jian Zhang, Fangxiang Mu, Zhongjie Guo, Zhuhua Cai, Xianghui Zeng, Lirong Du, Fang Wang

**Affiliations:** 1 Department of Reproductive Medicine, Lanzhou University Second Hospital, Lanzhou, 730030 China; 2 Obstetrics Department, First Affiliated Hospital of Chongqing Medical University, Chongqing, 400042 China; 3 Obstetrics Department, Third Hospital Affiliated to Guangdong Pharmaceutical University, Guangdong, 510410 China; 4 Gynaecology Department, Rui’an People’s Hospital, Wenzhou, 325207 China; 5 Department of Reproductive Medicine, Qinghai Provincial People’s Hospital, Xining, 810007 China; 6 Eugenics Clinical Department, Hebei Reproductive Health Hospital, Shijiazhuang, 050090 China

**Keywords:** Foetal chromosome karyotypes, Spontaneous abortion, Retrospective multicentre study

## Abstract

**Background:**

Abnormal foetal tissue chromosome karyotypes are one of the important pathogenic factors for spontaneous abortion (SA). To investigate the age and abnormal foetal karyotypes of 1903 couples who experienced SA.

**Methods:**

A retrospective multicentre study collected age and foetal tissue karyotypes CNV-seq data of 1903 SA couples from 6 hospitals in 5 regions from January 2017 to March 2022. The distribution and correlation of abnormal foetal tissue karyotypes were evaluated by using regions and age.

**Results:**

In our study, 1140 couples (60.5% of the total) had abnormal foetal tissue chromosome karyotypes in all regions. We found that there were differences in the number of abnormal foetal tissue chromosome karyotypes, of which the incidence of trisomy was higher. At the same time, the populations situated in the eastern region had a more triploid (15.5%) distribution, trisomy (58.1%) in the southern region, mosaicism (14.8%) and microduplication (31.7%) in the southwestern region, microdeletion (16.7%) in the northern region. There are variances across areas, and it is more common in the north. The incidence risk of prenatal chromosomal abnormalities varied according to age group.

**Conclusion:**

The findings of this study suggest that the karyotypes of patients with abnormal foetal tissue chromosome abortion in different regions were different. Meanwhile, patients ≥ 35 years old had a higher risk of abnormal foetal tissue chromosome abortion.

**Supplementary Information:**

The online version contains supplementary material available at 10.1186/s12884-023-06108-0.

Spontaneous abortion (SA) is one of the most prevalent complications during pregnancy. Early spontaneous abortion is a condition defined by pregnancy failure before 12 weeks of pregnancy [1]. It was reported that the risk of SA for women of reproductive age is approximately 10%. Only 3% to 11% of couples who experienced an early SA had a partner with chromosomal abnormalities [2–4]. Foetal tissue from couples with SA had a far higher frequency of chromosomal abnormalities than couples without SA. The current research indicates that foetal chromosomal abnormalities are still the most common cause of SA, accounting for 50% of cases or more [5, 6]. Previous studies have emphasized the need to study whether the demographic characteristics of patients are related to the causes of SA [7, 8].

Most studies have reported that the incidence and distribution of chromosome abnormalities in couples with SA are different in various countries and regions [9–18]. The incidence of them was less than 15%. The distribution of chromosome abnormalities among couples with abortion was mostly concentrated in the structure of the chromosomes (including translocation, inversion, duplication, insertion, and so on), the number of abnormal chromosomes was less, and the distribution of studies in various countries and regions was different [9, 11, 14, 18]. Compared with the probability of chromosomal abnormalities in couples with SA, the percentage of foetal chromosomal abnormalities is far higher in this part of the population. Chromosomal abnormalities in couples directly affect the foetal chromosomes, but even couples with normal chromosomes can miscarry due to the foetal chromosomal abnormalities [19]. There are many karyotypes including foetal chromosomal abnormalities, among which aneuploidy and polyploidy are common [20]. The age is a significant contributor to anomalies in foetal chromosomes [2, 6].

The purpose of this study was to investigate the age of 1903 couples with SA and to evaluate abnormal karyotypes among their foetal tissues in 5 regions of China (6 hospitals). We examined the correlation between the patient's age and karyotypes including foetal chromosome abnormalities. Additionally, we included the distribution of chromosomes and age in 5 regions.

## Materials and methods

### Patients

From January 2017 to March 2022, patients with SA were treated in 6 hospitals (Lanzhou University Second Hospital, Qinghai Provincial People's Hospital, Hebei Reproductive Health Hospital, Rui’an People's Hospital, Guangdong Pharmaceutical University Third Affiliated Hospital, and the First Affiliated Hospital of Chongqing Medical University) was taken as the research objects. The foetal tissue karyotypes copy number variation sequencing (CNV-seq) and age data of patients’ foetal tissue were collected in 6 hospitals. The inclusion criteria: 1. Early SA before 12 weeks of gestation; 2. The patients need to perform uterine cavity cleaning operations, and obtain abortion tissue for CNV-seq and provide examination report data for scientific research voluntarily; 3. The informed consent was signed by all patients in 6 hospitals. The study used non-identifiable patient data and was approved by the ethics review committee of the 6 hospitals. Studies were approved by the following ethical committees: the Ethics Committee of Rui'an People's Hospital, the Ethics Committee of Lanzhou University Second Hospital, the Ethics Committee of Qinghai Provincial People's Hospital, the Ethics Committee of First Affiliated Hospital of Chongqing Medical University, the Ethics Committee of Third Hospital Affiliated to Guangdong Pharmaceutical University, and the Ethics Committee of Hebei Reproductive Health Hospital. The research complies with the Declaration of Helsinki. To investigate regional differences, we divided the 6 hospitals into 5 regions for analysis and research. Lanzhou and Qinghai belong to Northwest China, Hebei belongs to North China, Rui’an belongs to East China, Guangdong belongs to South China, and Chongqing belongs to southwest China.

In this study, we focused on triploid, trisomy, mosaicism, 45,X, microduplication, microdeletion, and monosomy in the CNV-seq. Triploid refers to an abnormal condition in the number of chromosomes in a cell, where an increase in the number of chromosomes forms triploid, with each chromosome having three times the number of normal cells [21]. Trisomy refers to a chromosomal numerical abnormality where there is an extra copy of a chromosome compared to the normal cell complement. In trisomic cells, one of the chromosome pairs has an additional chromosome, resulting in three homologous chromosomes for that particular chromosome [22–24]. Mosaicism is a gene-related disease that refers to the presence of cell populations from different genomes in an organism [25, 26]. When the calculated copy number of CNV-seq is between 2.1 and 2.8, it indicates trisomic mosaicism, and between 1.2 and 1.9, there is monosomic/diploid mosaicism [27–29]. The majority (86.5%) of chimerism occurrence in CNV-seq is confined placental mosaicism (CPM, mosaicism occurs only in the placenta and not in the fetus), and a small portion (13.5%) is true fetal mosaicism (TFM, mosaicism occurs both in the placenta and fetus) [30–32]. A few common and well-known disease-causing rearrangements between the 30 kb and 5 Mb size-range, are referred to as chromosomal microdeletion and microduplication [33]. Monosomy refers to the presence of a single copy of a chromosome, representing a numerical aberration in the chromosome complement. In normal circumstances, chromosomes exist in pairs. However, chromosomal aberrations can lead to the presence of a monosomic chromosome, where only one copy of the chromosome is present [34–36]. A common example of chromosomal monosomy is Turner syndrome, also known as monosomy X (45,X) [37].

### Statistical analysis

The SPSS software (IBM, version 26.0) and R software (version 4.2.1) were used to calculate the data. The patients’ age and chromosome data were summarized by mean ± SD and proportions, and the data were compared across whole groups using chi-squared and Fisher exact test.

In order to better understand the relationship between age and abnormal foetal chromosomes, the ages of patients were divided by an optimal cutoff value determined using Youden’s index of the receiver operating characteristic curves (ROC). To test whether the effect of age on abnormal foetal chromosomes varied by region, statistical interaction terms were introduced into separate fully adjusted models (Adjustment for multiple comparisons: Bonferroni). Effect modification was tested using α = 0.10 threshold.

We presented risk ratios (RRs) and rate differences (RDs) with 95% confidence interval (95%CI) to compare foetal tissue chromosomal abnormalities-associated SA incidence rates in our study population with rates experienced by ages of SA patients in 5 regions. The RR of different categories of abnormal chromosome karyotypes was presented in different age groups by using forest plots. When the *p*-value was less than 0.05, the results were deemed statistically significant.

## Results

### Patient characteristics of 5 regions

From January 2017 to March 2022, of 1903 included patients with SA, the proportions of patients contributed by region were: East, 21.3% (405); North, 5.4% (103); Northwest, 15.3% (292); South, 22.7% (432); Southwest, 35.3% (671). Demographic and foetal tissue chromosome karyotype conditions were presented in Table 1. Of these, A greater proportion (60.5%, 1140/1903) was patients with abnormal foetal tissue chromosome karyotypes in all regions. The age was 30.9 ± 7.8 in all patients, the normal foetal tissue chromosome karyotypes’ mean age was 30.6 ± 4.5 and the abnormal was 30.9 ± 4.9. The most common number of abnormal chromosomes was one chromosome (One-chr, 83.5%, 952/1140) in foetal tissue chromosome karyotypes. 188 patients had ≥ 2 numbers and categories of abnormal foetal tissue chromosome karyotypes in the 1140 patients with abnormal foetal tissue chromosome karyotypes.

**Table 1:** Demographic and foetal tissue chromosome karyotypes of SA patients in 5 regions (6 hospitals)

Characteristic	All patients (*n* = 1903) ^1^	East (*n* = 405)	North (*n* = 103)	Northwest (*n* = 292)	South (*n* = 432)	Southwest (*n* = 671)	Test value^2^	*p* value^3^
Age	30.8 ± 4.7	29.2 ± 4.5 ^abcd^	31.3 ± 4.6	31.3 ± 4.4	32.2 ± 4.9 ^j^	30.6 ± 4.5	23.8	** < 0.001**
**Age of foetal tissue chromosome karyotypes**								
Normal	30.6 ± 4.4	28.9 ± 4.3 ^abcd^	31.1 ± 3.8	31.3 ± 4.2 h	31.6 ± 4.3 ^j^	30.0 ± 4.5	10.4	** < 0.001**
Abnormal	30.9 ± 4.9	29.4 ± 4.6 ^abcd^	31.4 ± 5.0	31.2 ± 4.5 ^hi^	32.9 ± 5.3 ^j^	30.8 ± 4.6	16.1	** < 0.001**
**Number of foetal tissue chromosome karyotypes**								
Normal	763(39.5)	145(35.8)	36(35.0)	146(50.0)	228(52.8)	208(31.0)	68.216	** < 0.001**
Abnormal	1140(60.5)	260(64.2) ^bc^	67(65.0)^ef^	146(50.0) ^i^	204(47.2) ^j^	463(69.0)		
**Number of abnormal foetal tissue chromosome karyotypes **^**4**^								
One-chr	952(83.5)	198(76.2) ^bd^	54(80.6) ^eg^	97(66.4) ^hi^	168(82.4)^j^	435(94.0)	78.385	** < 0.001**
Two-chr	76(6.7)	17(6.5) ^bd^	6(9.0) ^eg^	32(21.9) ^hi^	9(4.4)	12(2.5)	53.809	** < 0.001**
Multiple-chr	112(9.8)	45(17.3) ^d^	7(10.4) ^g^	17(11.6) ^i^	27(13.2) ^j^	16(3.5)	44.297	** < 0.001**
**Categories of abnormal foetal tissue chromosome karyotypes**								
** Number**	***n* = 1279**	***n* = 284**	***n* = 90**	***n* = 191**	***n* = 234**	***n* = 480**		
Triploid	102(8.0)	44(15.5) ^abd^	4(4.4)	9(4.7) ^h^	27(11.5) ^j^	18(3.8)	39.688	** < 0.001**
Trisomy	584(45.7)	164(57.7) ^bd^	43(47.8) ^g^	72(37.7) ^h^	136(58.1) ^j^	169(35.2)	57.539	** < 0.001**
Mosaicism	127(9.9)	13(4.6) ^abd^	11(12.2) ^f^	24(12.6) ^h^	8(3.4) ^j^	71(14.8)	37.791	** < 0.001**
45,X	87(6.8)	28(9.9) ^b^	8(8.9)	8(4.2)	13(5.6)	30(6.3)	7.409	0.112
Microduplication	260(20.3)	9(3.2) ^abcd^	9(10.0) ^eg^	58(30.4) ^h^	32(13.7) ^j^	152(31.7)	113.938	** < 0.001**
Microdeletion	92(7.2)	20(7.0) ^a^	15(16.7) ^fg^	17(8.9)	16(6.8)	24(5.0)	14.301	**0.006**
Monosomy	27(2.1)	6(2.1)	0(0.0)	3(1.6)	2(0.9) ^j^	16(3.3)	6.369	0.148

^1^ Summarized as number (percentage) or mean ± SD. ^2^Age’s analysis was using the ANOVA test, other indexes were using the Chi-square test or Fisher exact test. ^3^ The bold *p* value was statistically significant. ^4^ Multiple-chr: number of abnormal chromosomes ≥ 3

East vs. North ^a^ Northwest ^b^, South ^c^ Southwest ^d^ North vs. Northwest ^e^, South ^f^, Southwest ^g^, Northwest vs. South ^h^, Southwest ^I^, South vs. Southwest ^j^

The *p* value of comparison was statistically significant

**Fig. 1:**
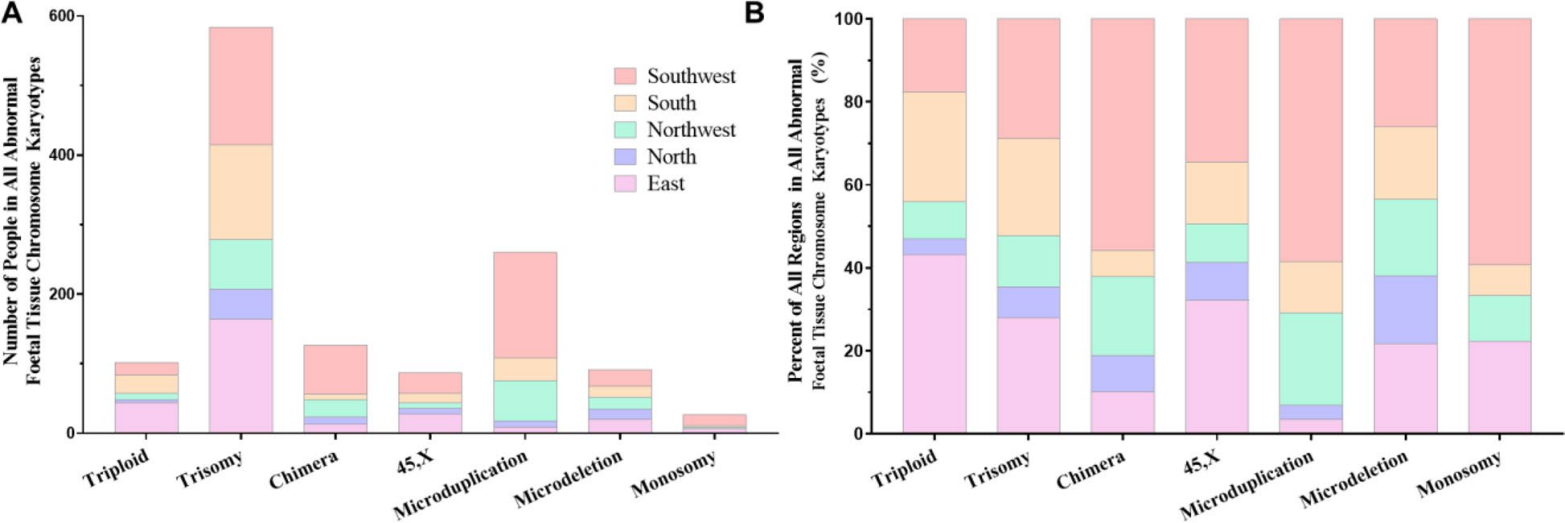
The stacked histogram of distribution for abnormal foetal tissue chromosome karyotypesin all regions. **A** The number of patients with all abnormal foetal tissue chromosome karyotypes. **B** The percentages of patients with all abnormal foetal tissue chromosome karyotypes

There were 1279 categories of abnormal foetal tissue chromosome karyotypes in 1140 patients. The number of patients in all abnormal foetal tissue chromosome karyotypes was presented in Table 1 and Fig. 1 (including the number and percentage stacked histogram). Categories of abnormal foetal tissue chromosome karyotypes were trisomy (45.7%, 584/1279), microduplication (20.3%, 260/1279), mosaicism (9.9%, 127/1279), triploid (8%, 102/1279), microdeletion (7.2%, 92/1279), 45,X (6.8%, 87/1279), and monosomy (2.1%, 27/1279). The most likely occurrence of abnormal foetal tissue chromosome karyotypes in different regions was different. The populations situated in the eastern region had a more triploid (15.5%, 44/284) distribution, trisomy (58.1%, 136/234) in the southern region, mosaicism (14.8%, 71/480), and microduplication (31.7%, 152/480) in the southwestern region, microdeletion (16.7%, 15/90) in the northern region. There was no significant difference in the frequency of 45,X and monosomy in each region.

### Distribution of abnormal chromosome karyotypes in 23 pairs of chromosomes

**Fig. 2:**
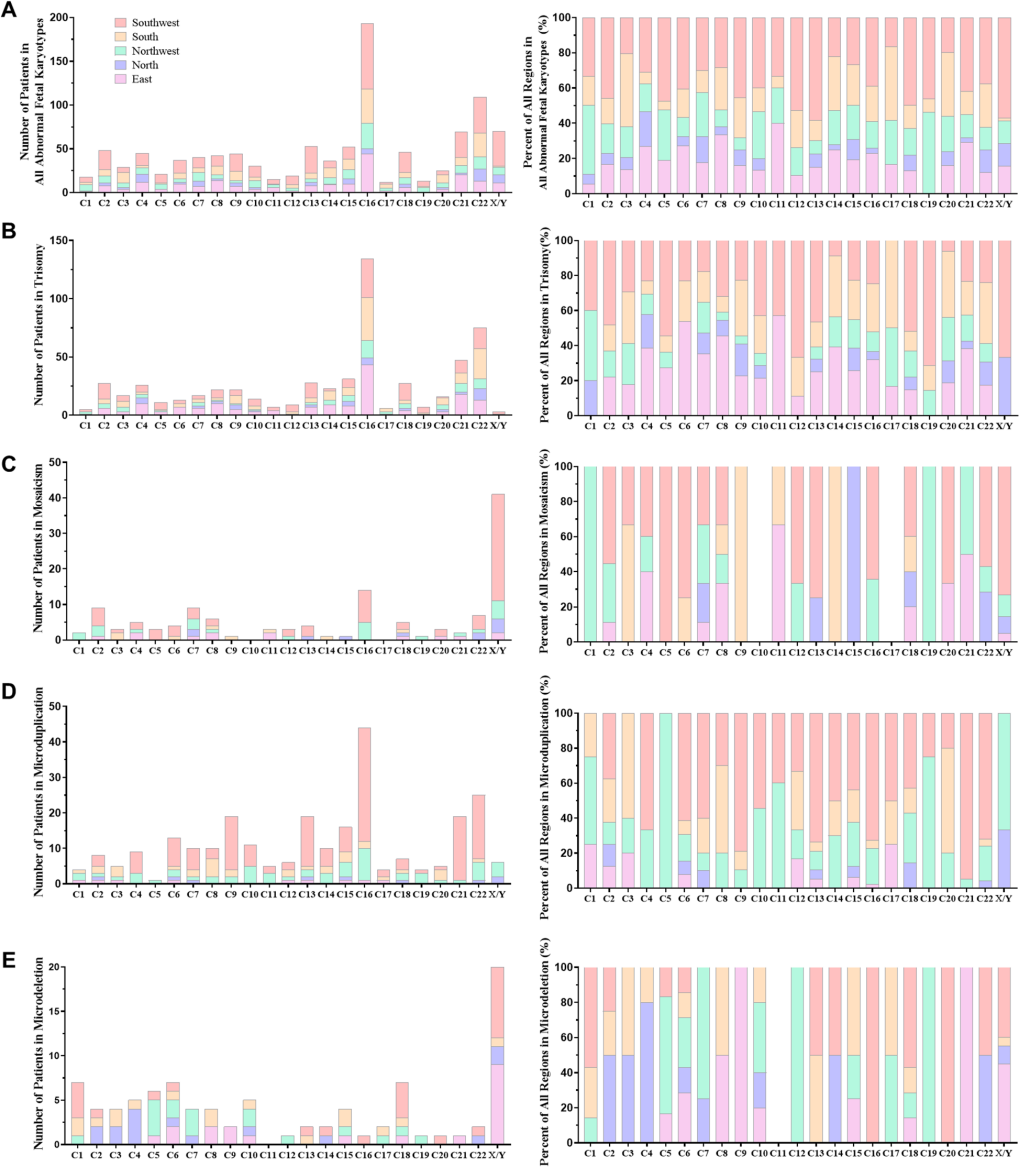
The stacked histogram of distribution forthe 23 pairs chromosomes by region in abnormal foetal tissue chromosome karyotypes. **A** The number and percentage of distribution for the 23 pairs chromosomes inabnormal foetal tissue chromosome karyotypes. **B** The distribution for the 23 pairs chromosomes intrisomy. **C** The distribution for the 23 pairs chromosomes inmosaicism. **D** The distribution for the 23 pairs chromosomes inmicroduplication. **E** The distribution for the 23 pairs chromosomes inmicrodeletion

The distribution of the 23 pairs of chromosomes by region was in Fig. 2 and Supplementary Table 1. The distribution of all abnormal foetal tissue chromosome karyotypes was enrichment on chromosomes 16 (18.1%, 193/1066) and 22 (10.2%, 109/1066), and fewest on chromosome 17 (1.1%, 12/1066). There was a statistical difference in the distribution of chromosomes 3, 4, 19, 22, and X/Y of all abnormal foetal tissue chromosome karyotypes in each region (*p* = 0.026, 0.007, 0.029, 0.018, and *p* < 0.001, respectively, Fig. 2A). The most common chromosome distribution of trisomy was not random with more enrichment on chromosomes 16 (22.9%, 134/584) and 22 (12.8%, 75/584), and fewest on chromosome X/Y (0.5%, 3/584). There was a statistical difference in the distribution of chromosome 22 of trisomy in each region (*p* = 0.011, Fig. 2B). The distribution of mosaicism was enrichment on chromosome X/Y (32.3%, 41/127) and 16 (11.0%, 14/127), and showed no signs on chromosome 10 (0%, 0/127) and 17 (0%, 0/127). There was a statistical difference in the distribution of chromosomes 3, 11, and X/Y of mosaicism in each region (*p* = 0.032, 0.007, 0.033, respectively, Fig. 2C). The distribution of microduplication was enrichment on chromosome 16 (16.9%, 44/260), and fewest on chromosome 5 (0.4%, 1/260). There was a statistical difference in the distribution of chromosomes 1, 3, 8, 21, and X/Y of microduplication in each region (*p* = 0.032, 0.003, 0.027, 0.036, 0.001, respectively, Fig. 2D). The distribution of microdeletion was enrichment on chromosome X/Y (21.7%, 20/92), and fewest on chromosome 11 (0%, 0/92). There was a statistical difference in the distribution of chromosomes 3, 4, 5, 7, and X/Y of microdeletion in each region (*p* = 0.043, 0.001, 0.049, 0.022, 0.002, respectively, Fig. 2E).

### Distribution condition of patients in different age groups and regions

**Fig. 3:**
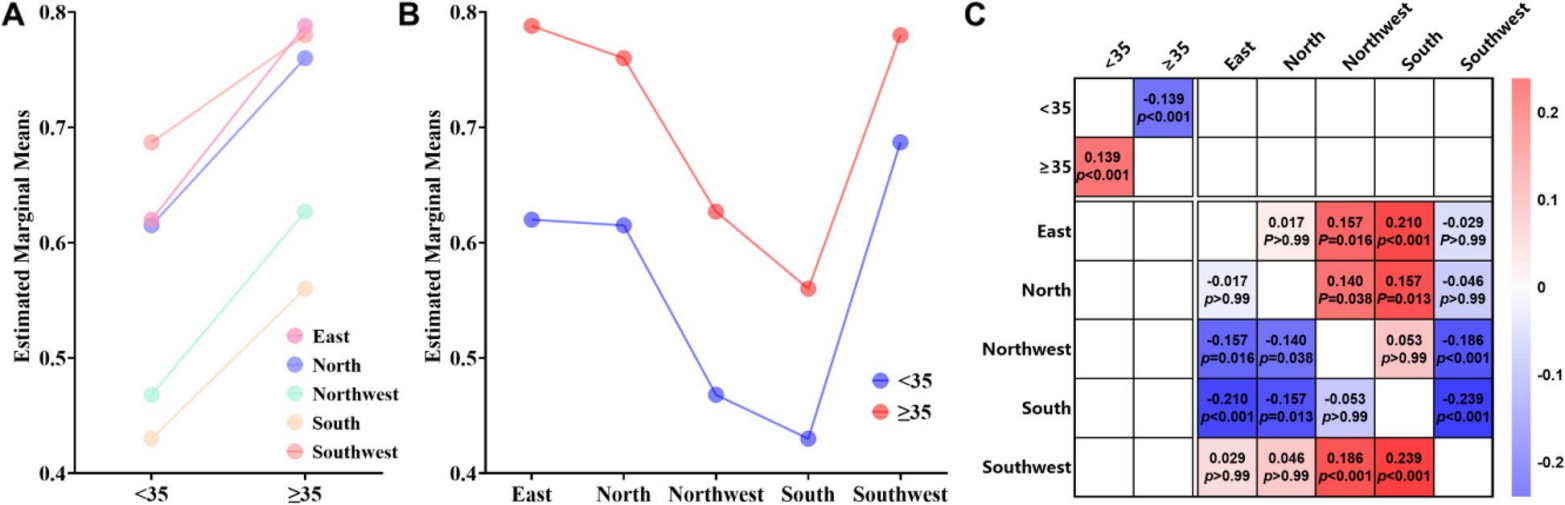
The main effect and interaction effect in age and regions. **A** The occurrence of abnormal foetal tissue chromosome karyotypes for 5 regions stratified byage. **B** The occurrence of abnormal foetal tissue chromosome karyotypes for < 35 and ≥ 35 years stratified by 5 regions. **C** The heatmap of main effect in age and regions. Red meant more abnormal foetal tissue chromosome karyotypes on the left than on the top. Blue meant the less on the left. One cell included mean difference value and *p*value. blank cells indicated that there was no significant interaction effectin age *region (*p* = 0.883)

As the single index, the optimal cut-off value of age was analyzed by ROC for grouping only. The AUC (95% CI) of age in all regions was 0.5 (0.5–0.6), *p* = 0.025. Based on the Youden index, the optimal cut-off for age was 34.5 years with sensitivity (24.4%) and specificity (82.7%). The patients were divided into the < 35 years group and the ≥ 35 years group. We evaluated whether the interaction between age and region was related to the occurrence of abnormal foetal tissue chromosome karyotypes (Fig. 3). Regardless of region, patients over 35 years old had more abnormal foetal tissue chromosome karyotypes than patients under 35 years old (Figs. 3A and B). There was a main effect of age (F = 18.4, *p* < 0.001, η2 = 0.009) and region (F = 15.8, *p* < 0.001, η2 = 0.032), but no interaction effect of age * region (F = 0.3, *p* value for interaction = 0.883, η2 = 0.001). The pairwise comparisons of the main effect in age and regions were shown in Fig. 3. The results revealed no interaction between age and regions, and the parallelism test was passed (*p* value for interaction = 0.883). The covariance analysis findings showed that age variations may cause considerable changes in the rate of foetal tissue chromosome abnormalities, and that when age was controlled for, the abnormality rate varies dramatically between regions. The covariance results indicate that age differences could lead to significant changes in the rate of foetal tissue chromosome abnormalities, and controlling for age, the abnormality rate varies significantly among different regions (Table 2). Compared with < 35 years patients, ≥ 35 years patients observed increases (mean difference = 0.139, *p* < 0.001). The patients in the eastern, north, and southwestern region were more SA with abnormal foetal tissue chromosome karyotypes than northwestern (mean difference = 0.157, 0.140, 0.186, *p* = 0.016, 0.038, < 0.001, respectively) and southern (mean difference = 0.210, 0.157, 0.239, *p* < 0.001, 0.013, < 0.001, respectively, Fig. 3C).

**Table 2:** The covariance analysis results of SA patients with normal and abnormal foetal tissue chromosome karyotypes by age in all regions

Variables	OR	95%CI	Z value	*p value*
Region (South) Ref	3.0	(1.5–5.9)	3.1	** < 0.001**
Region (East)	0.5	(0.3–0.7)	-3.3	** < 0.001**
Region (North)	0.9	(0.6–1.2)	-0.9	0.364
Region (Northwest)	0.5	(0.3–0.6)	-5.4	** < 0.001**
Region (Southwest)	0.4	(0.3–0.5)	-7.5	** < 0.001**

The region results of covariance analysis when maternal age was controlled. The bold *p* value was statistically significant

The proportion of ≥ 35 years patients was slightly higher than < 35 years (59.7 vs. 68.3%, Chi-square value = 10.6, *p* < 0.001). Overall, the relative risk rate (RR) of ≥ 35 years patients was a significant 1.3-fold higher than < 35 years in all regions (RR, 1.3, 95%CI, 1.1–1.5), equating to an absolute RD of 8.6% (95%CI, 6.4–10.3) (Table 3). A very similar situation was observed for each region but the northern region.

**Table 3:** TheriskofSApatients with normal and abnormal foetal tissue chromosome karyotypes by age in allregions

Region	Age	Abnormal	N	Chi-square	*p value*	Rate(%)	95%CI	RR	95%CI	RD%	95%CI
**All**	< 35	886	1484	10.6	** < 0.001**	59.7	(57.2–62.2)	Ref	(1.1–1.5)	Ref	(6.4–10.3)
	≥ 35	286	419			68.3	(63.6–72.5)	1.3		8.6	
**East**	< 35	219	353	5.6	**0.018**	62	(57.0–67.1)	Ref	(1.1–1.5)	Ref	(3.0–27.1)
	≥ 35	41	52			78.8	(67.7–89.9)	1.3		16.8	
**North**	< 35	48	78	1.8	0.187	61.5	(50.7–72.3)	Ref	(0.9–1.6)	Ref	(-7.4–31.2)
	≥ 35	19	25			76	(59.3–92.7)	1.2		14.5	
**Northwest**	< 35	109	233	4.8	**0.029**	46.8	(40.4–53.2)	Ref	(1.1–1.7)	Ref	(1.7–28.8)
	≥ 35	37	59			62.7	(50.4–75.1)	1.3		15.9	
**South**	< 35	125	291	6.5	**0.011**	43.0	(37.4–48.7)	Ref	(1.1–1.6)	Ref	(10.4–15.3)
	≥ 35	79	141			56.0	(47.8–64.0)	1.3		13.0	
**Southwest**	< 35	385	547	4.5	**0.038**	71.2	(67.2–74.8)	Ref	(0.9–1.7)	Ref	(0.9–7.9)
	≥ 35	99	124			76.2	(68.1–82.7)	1.2		5.0	

The bold *p* value was statistically significant

**Fig. 4:**
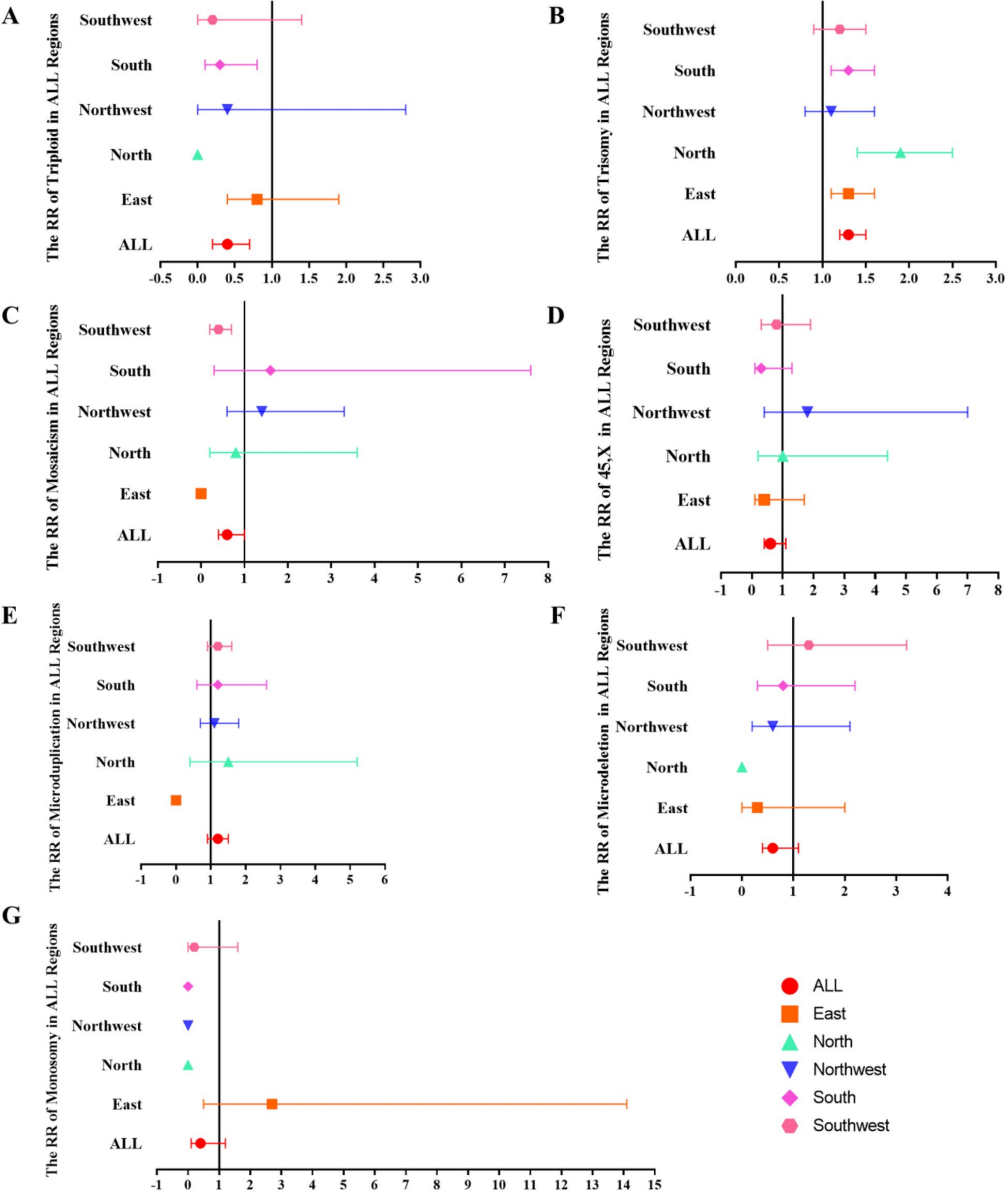
The forest plots forrisk of all categories of abnormal foetal tissue chromosome karyotypes in different age groups. **A** The forest plots for RR of triploid in all regions. **B** The forest plots for RR of trisomy in all regions. **C** The forest plots for RR of mosaicism in all regions. **D** The forest plots for RR of 45,X in all regions. **E** The forest plots for RR of microduplication in all regions. **F** The forest plots for RR of microdeletion in all regions. **G** The forest plots for RR of monosomyin all regions. The no-effect line was 1. The left side of no-effect line was < 35 years group, and right was ≥ 35 years group

We observed the risk of all categories of abnormal foetal tissue chromosome karyotypes in different age groups. Overall, the risk of triploid in ≥ 35 years patients was lower than that of < 35 years (RR, 0.4, 95%CI, 0.2–0.7, *p* = 0.031), while the risk of trisomy was a significant 1.2-fold higher in ≥ 35 years patients than that < 35 years (RR, 1.3, 95%CI, 1.2–1.5, *p* < 0.001). The risks of other abnormal foetal tissue chromosome karyotype categories were not statistically significant in the < 35 and ≥ 35 years groups of patients (*p* > 0.05, Table 4 and Fig. 4).

**Table 4:** The risk of all abnormal foetal tissue chromosome karyotypes by age in all regions

**Abnormal foetal tissue chromosome karyotypes**	**All**	**East**	**North**	**Northwest**	**South**	**Southwest**	**Z value**	***p value***
Triploid	0.4(0.2–0.7)	0.8(0.4–1.9)	0.0(0.0–0.0)	0.4(0.0–2.8)	0.3(0.1–0.8)	0.2(0.0–1.4)	2.16	**0.031**
Trisomy	1.3(1.2–1.5)	1.3(1.1–1.6)	1.9(1.4–2.5)	1.1(0.8–1.6)	1.3(1.1–1.6)	1.2(0.9–1.5)	5.33	** < 0.001**
Mosaicism	0.6(0.4–1.0)	0.0(0.0–0.0)	0.8(0.2–3.6)	1.4(0.6–3.3)	1.6(0.3–7.6)	0.4(0.2–0.7)	1.36	0.174
45,X	0.6(0.4–1.1)	0.4(0.1–1.7)	1.0(0.2–4.4)	1.8(0.4–7.0)	0.3(0.1–1.3)	0.8(0.3–1.9)	1.44	0.149
Microduplication	1.2(0.9–1.5)	0.0(0.0–0.0)	1.5(0.4–5.2)	1.1(0.7–1.8)	1.2(0.6–2.6)	1.2(0.9–1.6)	1.44	0.149
Microdeletion	0.6(0.4–1.1)	0.3(0.0–2.0)	0.0(0.0–0.0)	0.6(0.2–2.1)	0.8(0.3–2.2)	1.3(0.5–3.2)	0.52	0.604
Monosomy	0.4(0.1–1.2)	2.7(0.5–14.1)	0.0(0.0–0.0)	0.0(0.0–0.0)	0.0(0.0–0.0)	0.2(0.0–1.6)	0.29	0.771

The bold *p* value was statistically significant

## Discussion

Abnormal foetal tissue chromosome karyotypes have long been recognized as the major cause of SA, with abnormal foetal tissue chromosome karyotypes accounting for nearly half of all SAs [38]. The incidence of abnormal foetal tissue chromosome karyotypes differs geographically. Our study found that the incidence of abnormal foetal tissue chromosome karyotypes in most regions was more 50%, among which the incidence in the eastern, northern, and southwestern regions was more than 60%.

The region plays an important role in the pathogenesis of embryo chromosome abnormalities, but the research on embryo chromosomal abnormalities is different in different regions. It is unknown climate, living environment, eating habits, ethnic differences, and other regional factors affect the distribution of embryo chromosome abnormalities. At present, there are few reports on the relevant regional environments and abnormal chromosome distributions, and the karyotypes needs to be further studied. A study in Northeast China found that trisomy 22 and trisomy 16 were more prevalent, and the incidence of foetal tissue chromosomal abnormalities in pregnant women over 40 years old was significantly higher than that in other age groups [39]. According to Swedish research, trisomy 16 and sex chromosomal abnormalities accounted for a high proportion of all chromosomal abnormalities. The autosomal and X chromosomes were positively associated with the age, but the X single chromosome and polyploidy were negatively related to the age [40]. Korean research discovered a high prevalence of trisomy 22 but did not examine the association between age and chromosomal abnormalities [20]. Most previous studies reported foetal tissue chromosomal abnormalities, focusing on the overall distribution of the number of abnormal karyotypes in 23 pairs of chromosomes. Moreover, relatively few studies on the various regions and age distributions of patients with abnormal foetal tissue chromosome karyotypes have been reported in association with SA.

This study reviewed the foetal tissue chromosome karyotypes study of 1903 patients, who belong to our cohort study on SA. The present study encompassed patients within 5 defined geographic regions in China, without selection for age. The most frequent abnormal foetal tissue chromosome karyotype among SA patients was trisomy. The present study statistically validated that there were significant differences in the regional distribution depending on the abnormal karyotype, unlike the previously mentioned earlier study. In our study, nearly half of the patients had trisomy. The distribution of trisomy patients in the East and South was greater than that in the West and North. The distributions of other karyotypes (triploid, mosaicism, microduplication, microdeletion) were also different in different regions. We also confirmed that the incidence of foetal chromosomal abnormalities in SA patients over 35 years old was higher. At the same time, the karyotypes of abnormal embryos in different age groups were found to be different.

Triploid has been identified as a significant contributor to spontaneous abortion. It may have anything to do with the father's age [41]. Trisomy is one of major cause of spontaneous abortion. This chromosomal abnormality interferes with normal foetal development, leading to problems such as incomplete foetal growth, organ malformations, and functional impairments, ultimately resulting in the inability to sustain pregnancy or spontaneous abortion. It is important to note that not all cases of trisomy result in spontaneous abortion. Some trisomy abnormalities may lead to the birth of children with a range of genetic disorders and developmental disabilities rather than abortion during pregnancy [42, 43]. Research has shown that chromosomal abnormalities resulting from mosaicism may be associated with abnormal foetal development, thereby increasing the risk of spontaneous abortion [44, 45]. Beyond the risk of spontaneous abortion, CPM have a major clinical impact on foetal placental development and are detectable through noninvasive prenatal testing and chorion villous sampling. These include the risks of stunted foetal growth, small for gestational age, foetal growth restriction, and hypertensive disorders [30, 46, 47]. Microduplication and microdeletion have been linked to miscarriage, however their role in this phenomenon has been little studied [48, 49]. The most common single chromosome is 45, X, also known as Turner syndrome, and not all monosomy could cause miscarriage. 45, X mainly affects the reproduction, intelligence, and body development of the fetus [50, 51]. By clearing out these mysteries, we may advance towards more effective clinical and patient management.

### Strengths and limitations

This study’s major strength was its capture of SA patients with data on the foetal chromosomes from 5 regions across China (6 hospitals). A total of 1140 SA patients with abnormal foetal tissue chromosome karyotypes from 5 years in 6 hospitals were included, and disregarding patients’ abortion status enabled a more thorough understanding of distribution for abnormal foetal tissue chromosome karyotypes. In addition, we ascertained the age and regions considering the distribution of abnormal foetal tissue chromosome karyotypes and distinguished among the occurrence of categories and 23 pairs of chromosomes. Our study also had limitations, including the fact that more potential factors were not used for descriptive analysis in this study. For mosaicism, we could not distinguish between CPM and TFM.

## Conclusion

Overall, the findings of this study suggest that the incidence rate of SA among patients with abnormal foetal tissue chromosome karyotypes was more than half of patients with SA. In addition, correlations between the abnormal foetal tissue chromosome karyotypes and the patient's demographic data (age or region distribution) were observed. With a large cohort, we were able to provide a wide spectrum of data on the frequency and different types of chromosomal abnormalities. Thus, our study provides valuable data for a better understanding of chromosome analysis in couples experiencing SA.

### Supplementary Information

**Additional file 1: Supplementary Table 1.** The number of percentage of distribution for the 23 pairs chromosomes in abnormal fetal karyotypes. **Supplementary Table 2.** The distribution for the 23 pairs chromosomes in trisomy. **Supplementary Table 3.** The distribution for the 23 pairs chromosomes in trisomy mosaicism. **Supplementary Table 4.** The distribution for the 23 pairs of chromosomes in trisomy microduplication. **Supplementary Table 5.** The distribution for the 23 pairs chromosomes in trisomy microdeletion.

## Data Availability

The data that support the findings of this study are available on request from the corresponding author. Researchers who are interested in working together on our study are more than welcome to collaborate. Contact the paper's corresponding author, Fang Wang, [ery_fwang@lzu.edu.cn].
