# Supplementary material for: Chromosome analysis of foetal tissue from 1903 spontaneous abortion patients in 5 regions of China: a retrospective multicentre study
Source: BMC Pregnancy Childbirth. 2023 Nov 25;23:818. doi: 10.1186/s12884-023-06108-0 (PMC10675863; doi:10.1186/s12884-023-06108-0)
Supplement: Supplementary file 1 — Additional file 1: Supplementary Table 1. The number of percentage of distribution for the 23 pairs chromosomes in abnormal fetal karyotypes. Supplementary Table 2. The distribution for the 23 pairs chromosomes in trisomy. Supplementary Table 3. The distribution for the 23 pairs chromosomes in trisomy mosaicism. Supplementary Table 4. The distribution for the 23 pairs of chromosomes in trisomy microduplication. Supplementary Table 5. The distribution for the 23 pairs chromosomes in trisomy microdeletion. [file 12884_2023_6108_MOESM1_ESM.pdf]

Supplementary Tables

Supplementary Table 1 The number and percentage of distribution for the 23 pairs chromosomes in abnormal fetal karyotypes

	**All patients**	**East**	**North**	**Northwest**	**South**	**Southwest**	**fisher**	***p* value**
C1	18(1.7)	1(0.5)	1(1.3)	7(4.1)	3(1.6)	6(1.4)	6.578	0.125
C2	48(4.5)	8(3.9)	3(3.8)	8(4.7)	7(3.6)	22(5.3)	0.988	0.919
C3	29(2.7)	4(1.9)	2(2.5)	5(2.9)	12(6.3)	6(1.4)	10.393	**0.026**
C4	45(4.2)	12(5.8)	9(11.4)	7(4.1)	3(1.6)	14(3.3)	13.512	**0.007**
C5	21(2)	4(1.9)	0(0)	6(3.5)	1(0.5)	10(2.4)	5.558	0.199
C6	37(3.5)	10(4.9)	2(2.5)	4(2.3)	6(3.1)	15(3.6)	1.842	0.767
C7	40(3.8)	7(3.4)	6(7.6)	10(5.8)	5(2.6)	12(2.9)	6.621	0.144
C8	42(3.9)	14(6.8)	2(2.5)	4(2.3)	10(5.2)	12(2.9)	7.335	0.108
C9	44(4.1)	7(3.4)	4(5.1)	3(1.8)	10(5.2)	20(4.8)	4.18	0.372
C10	30(2.8)	4(1.9)	2(2.5)	8(4.7)	4(2.1)	12(2.9)	2.845	0.58
C11	15(1.4)	6(2.9)	0(0)	3(1.8)	1(0.5)	5(1.2)	4.646	0.277
C12	19(1.8)	2(1)	0(0)	3(1.8)	4(2.1)	10(2.4)	2.518	0.636
C13	53(5)	8(3.9)	4(5.1)	4(2.3)	6(3.1)	31(7.4)	8.982	0.055
C14	36(3.4)	9(4.4)	1(1.3)	7(4.1)	11(5.7)	8(1.9)	7.837	0.084
C15	52(4.9)	10(4.9)	6(7.6)	10(5.8)	12(6.3)	14(3.3)	5.019	0.276
C16	193(18.1)	44(21.4)	6(7.6)	29(17)	39(20.3)	75(17.9)	8.836	0.064
C17	12(1.1)	2(1)	0(0)	3(1.8)	5(2.6)	2(0.5)	5.909	0.139
C18	46(4.3)	6(2.9)	4(5.1)	7(4.1)	6(3.1)	23(5.5)	3.037	0.544
C19	13(1.2)	0(0)	0(0)	6(3.5)	1(0.5)	6(1.4)	9.102	**0.029**
C20	25(2.3)	4(1.9)	2(2.5)	5(2.9)	9(4.7)	5(1.2)	7.223	0.103
C21	69(6.5)	20(9.7)	2(2.5)	9(5.3)	9(4.7)	29(6.9)	6.588	0.153
C22	109(10.2)	13(6.3)	14(17.7)	14(8.2)	27(14.1)	41(9.8)	11.792	**0.018**
C23	70(6.6)	11(5.3)	9(11.4)	9(5.3)	1(0.5)	40(9.6)	26.035	**<0.001**

The bold *p* value was statistically significant.

Supplementary Table 2 The distribution for the 23 pairs chromosomes in trisomy.

	All patients	East	North	Northwest	South	Southwest	fisher	*p* value
**C1**	5(0.9)	0(0)	1(2.3)	2(2.8)	0(0)	2(1.2)	6.668	0.058
**C2**	27(4.6)	6(3.7)	0(0)	4(5.6)	4(2.9)	13(7.7)	6.237	0.16
**C3**	17(2.9)	3(1.8)	0(0)	4(5.6)	5(3.7)	5(3)	3.525	0.441
**C4**	26(4.5)	10(6.1)	5(11.6)	3(4.2)	2(1.5)	6(3.6)	8.803	0.05
**C5**	11(1.9)	3(1.8)	0(0)	1(1.4)	1(0.7)	6(3.6)	3.168	0.486
**Ci6**	13(2.2)	7(4.3)	0(0)	0(0)	3(2.2)	3(1.8)	4.296	0.311
**C7**	17(2.9)	6(3.7)	2(4.7)	3(4.2)	3(2.2)	3(1.8)	2.733	0.601
**C8**	22(3.8)	10(6.1)	2(4.7)	1(1.4)	2(1.5)	7(4.1)	5.48	0.212
**C9**	22(3.8)	5(3)	4(9.3)	1(1.4)	7(5.1)	5(3)	5.321	0.226
**C10**	14(2.4)	3(1.8)	1(2.3)	1(1.4)	3(2.2)	6(3.6)	1.396	0.873
**C11**	7(1.2)	4(2.4)	0(0)	0(0)	0(0)	3(1.8)	4.109	0.325
**C12**	9(1.5)	1(0.6)	0(0)	0(0)	2(1.5)	6(3.6)	4.852	0.224
**C13**	28(4.8)	7(4.3)	2(4.7)	2(2.8)	4(2.9)	13(7.7)	4.231	0.355
**C14**	23(3.9)	9(5.5)	0(0)	4(5.6)	8(5.9)	2(1.2)	8.331	0.06
**C15**	31(5.3)	8(4.9)	4(9.3)	5(6.9)	7(5.1)	7(4.1)	2.603	0.627
**C16**	134(22.9)	43(26.2)	6(14)	15(20.8)	37(27.2)	33(19.5)	5.532	0.236
**C17**	6(1)	1(0.6)	0(0)	2(2.8)	3(2.2)	0(0)	5.681	0.12
**C18**	27(4.6)	4(2.4)	2(4.7)	4(5.6)	3(2.2)	14(8.3)	8.298	0.065
**C19**	7(1.2)	0(0)	0(0)	1(1.4)	1(0.7)	5(3)	5.655	0.13
**C20**	16(2.7)	3(1.8)	2(4.7)	4(5.6)	6(4.4)	1(0.6)	8.309	0.052
**C21**	47(8)	18(11)	2(4.7)	7(9.7)	9(6.6)	11(6.5)	3.441	0.482
**C22**	75(12.8)	13(7.9)	9(23.3)	8(11.1)	26(19.1)	18(10.7)	12.86	**0.011**
**X/Y**	3(0.5)	0(0)	1(2.3)	0(0)	0(0)	2(1.2)	4.926	0.161

The bold *p* value was statistically significant.

Supplementary Table 3 The distribution for the 23 pairs chromosomes in trisomy mosaicism

	All patients	East	North	Northwest	South	Southwest	fisher	*p* value
**C1**	2(1.6)	0(0)	0(0)	2(8.3)	0(0)	0(0)	6.411	0.153
**C2**	9(7.1)	1(7.7)	0(0)	3(12.5)	0(0)	5(7)	1.784	0.764
**C3**	3(2.4)	0(0)	0(0)	0(0)	2(25)	1(1.4)	8.332	**0.032**
**C4**	5(3.9)	2(15.4)	0(0)	1(4.2)	0(0)	2(2.8)	4.111	0.24
**C5**	3(2.4)	0(0)	0(0)	0(0)	0(0)	3(4.2)	1.615	0.821
**C6**	4(3.1)	0(0)	0(0)	0(0)	1(12.5)	3(4.2)	3.205	0.389
**C7**	9(7.1)	1(7.7)	2(18.2)	3(12.5)	0(0)	3(4.2)	4.626	0.216
**C8**	6(4.7)	2(15.4)	0(0)	1(4.2)	1(12.5)	2(2.8)	5.281	0.141
**C9**	1(0.8)	0(0)	0(0)	0(0)	1(12.5)	0(0)	7.948	0.063
**C10**	0(0)	0(0)	0(0)	0(0)	0(0)	0(0)	*	*
**C11**	3(2.4)	2(15.4)	0(0)	0(0)	1(12.5)	0(0)	10.649	**0.007**
**C12**	3(2.4)	0(0)	0(0)	1(4.2)	0(0)	2(2.8)	1.529	1
**C13**	4(3.1)	0(0)	1(9.1)	0(0)	0(0)	3(4.2)	2.568	0.496
**C14**	1(0.8)	0(0)	0(0)	0(0)	1(12.5)	0(0)	7.948	0.063
**C15**	1(0.8)	0(0)	1(9.1)	0(0)	0(0)	0(0)	7.311	0.15
**C16**	14(11)	0(0)	0(0)	5(20.8)	0(0)	9(12.7)	4.683	0.239
**C17**	0(0)	0(0)	0(0)	0(0)	0(0)	0(0)	*	*
**C18**	5(3.9)	1(7.7)	1(9.1)	0(0)	1(12.5)	2(2.8)	5.096	0.179
**C19**	1(0.8)	0(0)	0(0)	1(4.2)	0(0)	0(0)	5.75	0.441
**C20**	3(2.4)	1(7.7)	0(0)	0(0)	0(0)	2(2.8)	2.756	0.65
**C21**	2(1.6)	1(7.7)	0(0)	1(4.2)	0(0)	0(0)	6.166	0.192
**C22**	7(5.5)	0(0)	2(18.2)	1(4.2)	0(0)	4(5.6)	3.317	0.401
**C23**	41(32.3)	2(15.4)	4(36.4)	5(20.8)	0(0)	30(42.3)	10.043	**0.033**

The bold *p* value was statistically significant.

Supplementary Table 4 The distribution for the 23 pairs chromosomes in trisomy microduplication

	All patients	East	North	Northwest	South	Southwest	fisher	*p* value
**C1**	4(1.5)	1(11.1)	0(0)	2(3.4)	1(3.1)	0(0)	9.961	**0.032**
**C2**	8(3.1)	1(11.1)	1(11.1)	1(1.7)	2(6.3)	3(2)	7.016	0.087
**C3**	5(1.9)	1(11.1)	0(0)	1(1.7)	3(9.4)	0(0)	13.568	**0.003**
**C4**	9(3.5)	0(0)	0(0)	3(5.2)	0(0)	6(3.9)	1.466	0.762
**C5**	1(0.4)	0(0)	0(0)	1(1.7)	0(0)	0(0)	6.523	0.415
**C6**	13(5)	1(11.1)	1(11.1)	2(3.4)	1(3.1)	8(5.3)	2.977	0.486
**C7**	10(3.8)	0(0)	1(11.1)	1(1.7)	2(6.3)	6(3.9)	3.252	0.407
**C8**	10(3.8)	0(0)	0(0)	2(3.4)	5(15.6)	3(2)	9.342	**0.027**
**C9**	19(7.3)	0(0)	0(0)	2(3.4)	2(6.3)	15(9.9)	2.588	0.545
**C10**	11(4.2)	0(0)	0(0)	5(8.6)	0(0)	6(3.9)	3.387	0.375
**C11**	5(1.9)	0(0)	0(0)	3(5.2)	0(0)	2(1.3)	3.732	0.457
**C12**	6(2.3)	1(11.1)	0(0)	1(1.7)	2(6.3)	2(1.3)	6.263	0.133
**C13**	19(7.3)	1(11.1)	1(11.1)	2(3.4)	1(3.1)	14(9.2)	3.719	0.392
**C14**	10(3.8)	0(0)	0(0)	3(5.2)	2(6.3)	5(3.3)	1.49	0.768
**C15**	16(6.2)	1(11.1)	1(11.1)	4(6.9)	3(9.4)	7(4.6)	3.6	0.383
**C16**	44(16.9)	1(11.1)	0(0)	9(15.5)	2(6.3)	32(21.1)	5.73	0.184
**C17**	4(1.5)	1(11.1)	0(0)	0(0)	1(3.1)	2(1.3)	6.085	0.149
**C18**	7(2.7)	0(0)	1(11.1)	2(3.4)	1(3.1)	3(2)	3.803	0.339
**C19**	4(1.5)	0(0)	0(0)	3(5.2)	0(0)	1(0.7)	5.385	0.197
**C20**	5(1.9)	0(0)	0(0)	1(1.7)	3(9.4)	1(0.7)	7.915	0.063
**C21**	19(7.3)	0(0)	0(0)	1(1.7)	0(0)	18(11.8)	9.217	**0.036**
**C22**	25(9.6)	0(0)	1(11.1)	5(8.6)	1(3.1)	18(11.8)	2.704	0.541
**C23**	6(2.3)	0(0)	2(22.2)	4(6.9)	0(0)	0(0)	16.804	**0.001**

The bold *p* value was statistically significant.

Supplementary Table 5 The distribution for the 23 pairs chromosomes in trisomy microdeletion

	**All patients**	**East**	**North**	**Northwest**	**South**	**Southwest**	**fisher**	***p* value**
**C1**	7(7.6)	0(0)	0(0)	1(5.9)	2(12.5)	4(16.7)	5.306	0.166
**C2**	4(4.3)	0(0)	2(13.3)	0(0)	1(6.3)	1(4.2)	3.879	0.278
**C3**	4(4.3)	0(0)	2(13.3)	0(0)	2(12.5)	0(0)	6.206	**0.043**
**C4**	5(5.4)	0(0)	4(26.7)	0(0)	1(6.3)	0(0)	9.994	**0.001**
**C5**	6(6.5)	1(5)	0(0)	4(23.5)	0(0)	1(4.2)	6.745	**0.049**
**C6**	7(7.6)	2(10)	1(6.7)	2(11.8)	1(6.3)	1(4.2)	1.453	0.899
**C7**	4(4.3)	0(0)	1(6.7)	3(17.6)	0(0)	0(0)	6.628	**0.022**
**C8**	4(4.3)	2(10)	0(0)	0(0)	2(12.5)	0(0)	5.019	0.104
**C9**	2(2.2)	2(10)	0(0)	0(0)	0(0)	0(0)	4.27p	0.132
**C10**	5(5.4)	1(5)	1(6.7)	2(11.8)	1(6.3)	0(0)	3.199	0.513
**C11**	0(0)	0(0)	0(0)	0(0)	0(0)	0(0)	-	-
**C12**	1(1.1)	0(0)	0(0)	1(5.9)	0(0)	0(0)	4.191	0.522
**C13**	2(2.2)	0(0)	0(0)	0(0)	1(6.3)	1(4.2)	2.863	0.788
**C14**	2(2.2)	0(0)	1(6.7)	0(0)	0(0)	1(4.2)	2.992	0.696
**C15**	4(4.3)	1(5)	0(0)	1(5.9)	2(12.5)	0(0)	3.856	0.322
**C16**	1(1.1)	0(0)	0(0)	0(0)	0(0)	1(4.2)	3.501	1
**C17**	2(2.2)	0(0)	0(0)	1(5.9)	1(6.3)	0(0)	3.553	0.315
**C18**	7(7.6)	1(5)	0(0)	1(5.9)	1(6.3)	4(16.7)	3.345	0.501
**C19**	1(1.1)	0(0)	0(0)	1(5.9)	0(0)	0(0)	4.191	0.522
**C20**	1(1.1)	0(0)	0(0)	0(0)	0(0)	1(4.2)	3.501	1
**C21**	1(1.1)	1(5)	0(0)	0(0)	0(0)	0(0)	3.866	0.739
**C22**	2(2.2)	0(0)	1(6.7)	0(0)	0(0)	1(4.2)	2.992	0.696
**C23**	20(21.7)	9(45)	2(13.3)	0(0)	1(6.3)	8(33.3)	15.577	**0.002**

The bold *p* value was statistically significant.
